# Eryptosis in Acute Patients: A Hypothesis on Its Potential Clinical Impact and Current Gaps in Evidence

**DOI:** 10.3390/cimb48010065

**Published:** 2026-01-06

**Authors:** Grazia Maria Virzì, Anna Clementi, Monica Zanella, Claudio Ronco

**Affiliations:** 1 Department of Nephrology, Dialysis and Transplant, St Bortolo Hospital, 36100 Vicenza, Italy; 2 IRRIV—International Renal Research Institute Vicenza-Foundation, 36100 Vicenza, Italy; 3 Department of Nephrology and Dialysis, Santa Marta and Santa Venera Hospital, 95024 Acireale, Italy

**Keywords:** eryptosis, AKI, sepsis, endotoxin

## Abstract

Sepsis is a life-threatening condition driven by a dysregulated host response to infection and remains a leading cause of mortality and morbidity worldwide. Among the many mechanisms implicated in its pathophysiology, the contribution of eryptosis—programmed red blood cell (RBC) death—has been suggested but remains insufficiently investigated. In this hypothesis paper, we propose that eryptosis may represent an underrecognized driver of sepsis-induced anemia and a potential contributor to subsequent organ dysfunction. This hypothesis is supported only by fragmented, predominantly preclinical evidence, which is currently too limited to allow firm conclusions. In this context, we critically revisit the sparse data linking sepsis, endotoxemia, and eryptosis, and outline a testable framework for understanding their possible interaction. We emphasize the substantial gaps in current knowledge, including the absence of robust clinical studies, the heterogeneity of existing experimental models, and persistent uncertainty regarding causality versus mere association. We also explore the theoretical implications of modulating eryptosis as a potential therapeutic approach. Our aim is to stimulate scientific discussion and promote targeted research efforts that may help determine whether addressing eryptosis could ultimately contribute to mitigating anemia, reducing organ injury, and improving outcomes in critically ill patients affected by sepsis.

## 1. Introduction

Erythrocytes, or red blood cells (RBCs), are vital components of vertebrate blood and constitute the most numerous cell type, making up about 45% of human blood volume. Unlike typical nucleated cells, mature RBCs are highly specialized: they are densely packed with hemoglobin and lack both a nucleus and organelles. These features, combined with their flexible, biconcave shape, enhance their ability to perform their main role—facilitating gas exchange. Measuring approximately 7–8 µm in diameter, RBCs are structurally adapted to efficiently transport oxygen and carbon dioxide throughout the body [1,2,3,4,5].

The structure of the RBC membrane is a paradigm of biological adaptability, characterized by a highly specialized and finely organized composition of proteins and lipids that grants it exceptional deformability and stability, simultaneously enabling complex interactions with, and critical responses to, a continuum of insults ranging from external (xenobiotic) compounds to circulating internal (endogenous) factors like inflammatory mediators [1,2,3,6,7,8]. Maintaining blood quality requires that aged or damaged red blood cells (RBCs) be efficiently removed and recycled through extravascular hemolysis, a function predominantly managed by the reticuloendothelial system (RES), which includes filtering organs like the spleen, bone marrow, and liver. Of these, the liver holds a central position in the removal pathway, utilizing Kupffer cells (KCs)—the tissue-resident macrophages—to identify specific molecular changes on the surface of senescent or impaired RBCs, leading to their subsequent recognition and phagocytosis [9].

RBCs undergo a form of programmed cell death known as eryptosis, which is functionally similar to apoptosis but uniquely adapted to the structure and physiology of RBCs. Unlike apoptosis, which occurs in nucleated cells and involves complex signaling pathways mediated by organelles such as mitochondria, eryptosis is specific to anucleate RBCs that lack such organelles. Eryptosis is initiated by a variety of molecular triggers, including intracellular Ca^2+^ overload, oxidative stress, energy depletion, hyperosmotic shock, and activation of Ca^2+^-permeable cation channels, which converge on membrane phosphatidylserine externalization and cytoskeletal destabilization [10]. Eryptosis is characterized by a well-defined set of features, including cell shrinkage, membrane blebbing, and externalization of phosphatidylserine (PS) on the cell surface. The exposure of PS serves as a key signal that tags the erythrocyte for removal. These PS-exposing RBCs can adhere to endothelial cells lining the blood vessels and are quickly recognized by macrophages, which interpret the exposed PS as an “eat me” signal. Macrophages then engulf and eliminate these altered cells. This efficient clearance mechanism prevents the accumulation of damaged or dysfunctional RBCs, thereby protecting the body from potential complications. Ultimately, eryptosis plays an essential role in preserving circulatory system health by ensuring the timely and non-inflammatory removal of senescent or impaired red blood cells [10,11,12]. This process plays a crucial role in maintaining blood homeostasis by removing defective, damaged, or aged RBCs from circulation in a controlled and non-inflammatory manner [6,7,8,13] and it is totally different from hemolysis [14]. Hemolysis is the rupture or destruction of red blood cells (RBCs), resulting in the release of hemoglobin and other intracellular contents, including lactate dehydrogenase, potassium, and various enzymes, into the surrounding plasma. Hemolysis can occur intravascularly, within the blood vessels, or extravascularly, primarily in the spleen and liver, where macrophages phagocytose damaged or senescent erythrocytes. Intravascular hemolysis typically results from mechanical trauma (such as prosthetic heart valves or extracorporeal circuits), immune-mediated processes (including complement activation or autoimmune hemolytic anemia), infections, oxidative stress, or exposure to certain toxins and drugs. Extravascular hemolysis, on the other hand, is most often due to the recognition and clearance of abnormal or opsonized RBCs by the reticuloendothelial system. When hemolysis becomes excessive, it can lead to a range of clinical complications. The loss of functional erythrocytes contributes to anemia, reducing oxygen-carrying capacity and potentially causing fatigue, dyspnea, and impaired tissue perfusion. The liberation of free hemoglobin into plasma can bind nitric oxide, leading to vasoconstriction, endothelial dysfunction, and increased risk of thrombosis. Additionally, the accumulation of heme and iron promotes oxidative stress and inflammation, further exacerbating tissue injury. The breakdown of hemoglobin produces bilirubin, which may accumulate and result in jaundice and, in severe cases, contribute to pigment nephropathy and acute kidney injury. Laboratory findings typically include elevated plasma-free hemoglobin, increased lactate dehydrogenase, decreased haptoglobin levels, and the presence of hemoglobinuria [15,16,17].

Eryptosis can be triggered by a variety of factors, including elevated intracellular calcium (Ca^2+^) levels, oxidative stress, inflammation, and several uremic toxins, each acting through distinct molecular pathways. Crucially, each of these factors initiates eryptosis by acting through distinct, though often overlapping, molecular pathways. In particular, increased intracellular Ca^2+^ induces PS exposure by activating Ca^2+^-dependent scramblases and simultaneously inhibiting aminophospholipid translocases (flippases), thereby disrupting membrane phospholipid asymmetry and promoting the externalization of PS to the outer leaflet of the RBC membrane [6,8,11].

This form of programmed red blood cell death is associated with, and contributes to, a wide range of clinical conditions such as anemia, metabolic syndrome, diabetes, uremia, sepsis, fever, and dehydration [6,10,18,19,20,21,22]. Recently, increasing attention has been given to the role of eryptosis in chronic kidney disease (CKD) and in patients undergoing chronic renal replacement therapies, such as peritoneal dialysis and hemodialysis, due to its contribution to anemia and kidney disease progression [23].

## 2. Aim of the Mini-Review

Enhanced eryptosis has been implicated in several acute clinical conditions, yet its precise role remains insufficiently defined. Although the majority of studies on eryptosis have been conducted in chronic kidney disease or general inflammatory conditions, the molecular pathways involved—oxidative stress, Ca^2+^ influx, and inflammatory cytokine signaling—are also activated in acute kidney injury. Nevertheless, extrapolation of these findings to acute patients should be done cautiously, and dedicated in vivo and ex vivo studies are required to confirm these mechanisms in acute settings.

Given the limited and largely indirect evidence available, the aim of this hypothesis-driven work is not to provide a comprehensive review, but rather to explore the potential relationship between sepsis, endotoxemia, and eryptosis by examining the mechanistic links that may connect these processes. Specifically, we propose the hypothesis that accelerated red blood cell death could contribute to the progression and severity of critical illness in the context of systemic infection and inflammatory burden. A further objective is to critically assess whether modulating eryptosis might represent a plausible therapeutic concept—while acknowledging that current data are too fragmented to support firm conclusions. By outlining this hypothesis, identifying major gaps in evidence, and highlighting areas where assumptions remain speculative, we aim to stimulate scientific discussion and guide future studies that may clarify whether targeting eryptosis could meaningfully influence outcomes in sepsis and associated complications.

## 3. Acute Kidney Injury (AKI) Scenarios

Acute kidney injury (AKI) is a rapid and abrupt decline in renal function, typically indicated by a rise in serum creatinine or a reduction in urine output. It can result from a variety of causes, such as ischemia, exposure to nephrotoxic agents, infections, sepsis, trauma, or pre-existing chronic kidney conditions. AKI is a serious condition that requires timely recognition and intervention to minimize kidney damage and prevent complications. The severity of AKI can vary, and if inadequately managed, it may progress to chronic kidney disease (CKD) [24,25,26,27]. Epidemiological studies report that AKI affects up to 30–50% of patients admitted to intensive care units, with an in-hospital mortality ranging from 20% to 60%, depending on severity and the need for renal replacement therapy. Moreover, survivors of AKI are at increased risk of developing chronic kidney disease, progression to end-stage kidney disease, recurrent hospitalizations, and long-term cardiovascular complications, highlighting its profound morbidity burden [28,29]. The risk of mortality increases particularly in the case of sepsis-associated AKI [30].

Timely recognition and proper management are essential to improving outcomes in patients with AKI. Moreover, AKI is a frequent and serious complication among critically ill individuals, often necessitating intensive care support. In the intensive care unit (ICU), acute kidney injury (AKI) requires vigilant monitoring and prompt treatment, given its association with increased morbidity, mortality, and extended hospitalization [31]. Management typically focuses on maintaining fluid and electrolyte homeostasis, treating the underlying cause, and, in more severe cases, may necessitate renal replacement therapy (RRT), such as continuous renal replacement therapy (CRRT), to support kidney function and improve clinical outcomes [32].

Eryptosis is increasingly recognized as a key contributor to the development of various renal disorders, especially AKI. In AKI, this form of eryptosis significantly contributes to erythrocyte dysfunction and early clearance, promoting anemia and aggravating kidney injury. The activation of eryptosis in this setting is believed to result from a multifaceted interaction of common AKI-related factors, including oxidative stress, the buildup of uremic toxins, and systemic inflammation. These elements not only cause direct damage to RBCs but also intensify the inflammatory milieu, establishing a self-perpetuating cycle that further compromises renal function [33,34,35].

Eryptosis in AKI also leads to the release of damage-associated molecular patterns (DAMPs), which amplify inflammation and tissue damage. Moreover, the exposure of PS on the surface of red blood cells facilitates their clearance by macrophages, worsening the inflammatory response and promoting endothelial dysfunction. This intricate interplay among eryptosis, oxidative stress, inflammation, and uremic toxins highlights the pivotal role of eryptosis in AKI pathogenesis and suggests its potential utility as both a biomarker and a therapeutic target in kidney injury management [5,6,34].

Currently, there is a limited number of studies that directly and systematically examine the causal relationship between AKI and eryptosis. A potential experimental approach to directly test whether AKI induces eryptosis would combine established in vivo models of AKI with quantitative assessment of eryptosis markers—such as PS exposure, cell shrinkage, and Ca^2+^ influx—in circulating RBCs, together with complementary ex vivo experiments exposing healthy erythrocytes to plasma from different AKI models. However, ad hoc in vivo and in vitro studies specifically designed to address this question are required to establish causality. For example, ischemia–reperfusion and nephrotoxic AKI models trigger eryptosis through overlapping but distinct mechanisms. Ischemia–reperfusion primarily induces oxidative stress and Ca^2+^ overload due to transient hypoxia and subsequent reoxygenation, leading to reactive oxygen species generation and membrane damage. In contrast, nephrotoxic agents, such as cisplatin or gentamicin, directly induce erythrocyte membrane injury, mitochondrial dysfunction, and oxidative stress, often accompanied by ATP depletion. Thus, while both pathways converge on PS exposure and cell shrinkage, the upstream molecular triggers and kinetics of eryptosis differ between the two models [5,7].

While eryptosis is increasingly acknowledged as a significant and potentially underappreciated factor in the development and progression of several kidney diseases, including but not limited to AKI, specific research efforts that rigorously detail and characterize the exact mechanistic and temporal connection between these conditions remain scarce and fragmented. Most of the available evidence is derived from indirect observations, small-scale experimental models, or extrapolations from studies focused on chronic kidney disease (CKD) and other inflammatory disorders, rather than from well-designed clinical trials or large observational cohorts dedicated specifically to investigating eryptosis in the context of AKI. Much of the existing evidence relies primarily on indirect observations rather than direct experimental data. Regrettably, there is a scarcity of direct scientific evidence supporting the involvement of eryptosis in AKI, and no research has specifically examined the connection between eryptosis and therapies such as CRRT or extracorporeal membrane oxygenation (ECMO) [5]. Although eryptosis has been well studied in chronic kidney disease (CKD) and other conditions characterized by oxidative stress and inflammation, its precise role in AKI pathophysiology remains largely uninvestigated [5,36,37].

**Figure 1:**
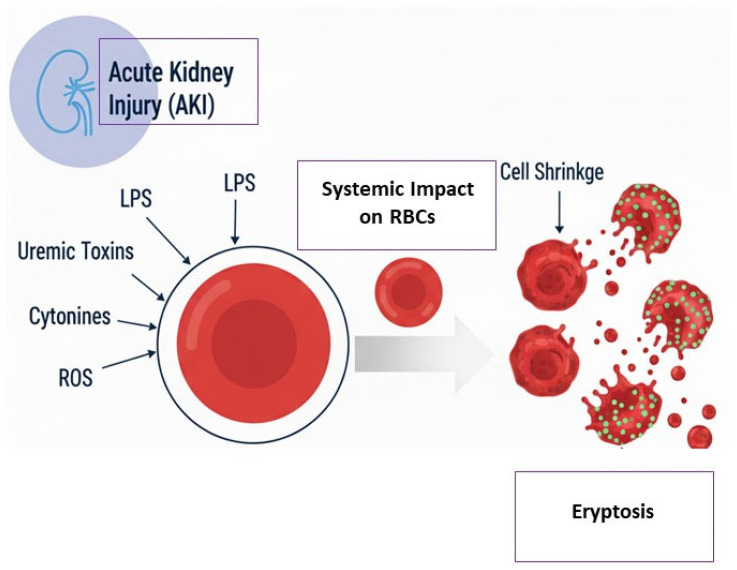
Schematic representation of the link between AKI and Eryptosis.

Furthermore, despite the critical importance of CRRT and ECMO in managing critically ill patients, the potential impact of these treatments on erythrocyte death via eryptosis has yet to be explored in the scientific literature. These life-sustaining therapies, while indispensable, expose blood components to artificial surfaces, mechanical stress, and pro-inflammatory environments, all of which could potentially modulate red blood cell viability [38]. Extracorporeal therapies such as CRRT and ECMO expose erythrocytes to non-physiological shear stress, artificial surfaces, and variable hemocompatibility, all of which can contribute to oxidative stress and perturbations in intracellular Ca^2+^ homeostasis. These factors may theoretically promote eryptosis by enhancing phosphatidylserine exposure and membrane destabilization. Although direct clinical evidence linking these modalities to eryptosis is currently limited, understanding these mechanistic intersections could inform strategies to minimize RBC damage during extracorporeal support. Yet, the extent to which such extracorporeal procedures influence the onset or progression of eryptosis is still unknown. To gain a more comprehensive and mechanistic understanding of the relationship between AKI and eryptosis, targeted research efforts are needed to investigate how key pathophysiological factors—such as systemic inflammation, uremic toxin accumulation, oxidative stress, and endothelial dysfunction—contribute to eryptosis during the course of AKI (Figure 1). Systemic inflammation in AKI contributes to Eryptosis by amplifying oxidative stress, promoting cytokine-mediated membrane damage, and sensitizing RBCs to injury. Pro-inflammatory cytokines such as TNF-α and IL-6 can enhance Ca^2+^ influx and reactive oxygen species generation in RBCs, accelerating PS exposure and cell shrinkage. Thus, inflammation acts as a key cofactor that potentiates eryptosis in the context of AKI [5]. In a similar way, ROS act as key mediators of eryptosis by damaging RBC membranes, oxidizing proteins and lipids, and activating Ca^2+^-permeable cation channels. The resulting Ca^2+^ influx, combined with oxidative damage, triggers membrane PS exposure, cell shrinkage, and ultimately eryptosis. ROS can therefore both initiate and amplify eryptosis, particularly under conditions of oxidative stress such as AKI or CKD [18,34].

It is also essential to determine whether the activation of eryptosis plays a causative role in exacerbating kidney damage or in perpetuating complications such as anemia, impaired oxygen delivery, and microcirculatory dysfunction [39,40]. Advancing knowledge in this area could open new avenues for therapeutic intervention aimed at preventing or modulating eryptosis, with the potential to improve clinical outcomes in AKI. This could be particularly relevant for refining and personalizing CRRT strategies, optimizing membrane biocompatibility, minimizing hemolytic burden, and ultimately enhancing renal recovery and patient survival in the most severe clinical scenarios.

## 4. Sepsis and Endotoxin-Mediated Responses

As previously noted, sepsis—a severe and potentially fatal condition marked by widespread infection and an excessive inflammatory response—is a common cause of AKI in critically ill patients. Sepsis is associated with substantial hospital expenses, increased morbidity and mortality, as well as high rates of hospitalization and inpatient deaths [31,32,33]. Recent research has increasingly examined the connection between sepsis and eryptosis. Sepsis can induce eryptosis in red blood cells, contributing to anemia and worsening organ dysfunction. Gaining a deeper understanding of this link is important, as it could reveal new therapeutic opportunities to enhance the treatment of sepsis-related AKI and other complications seen in critically ill patients. The development and progression of sepsis involve a complex and multifaceted interplay of biological processes [41]. These include infection, inflammation, oxidative stress, immune system impairment, coagulation disturbances, endothelial injury, tissue damage, and elevated rates of cell death or apoptosis. Together, these mechanisms operate at the tissue, cellular, and molecular levels, resulting in a widespread and detrimental systemic response [42]. In recent years, there has been an increasing emphasis on molecular and cellular investigations of sepsis, aimed at elucidating its precise underlying biological mechanisms [43,44,45,46].

**Figure 2:**
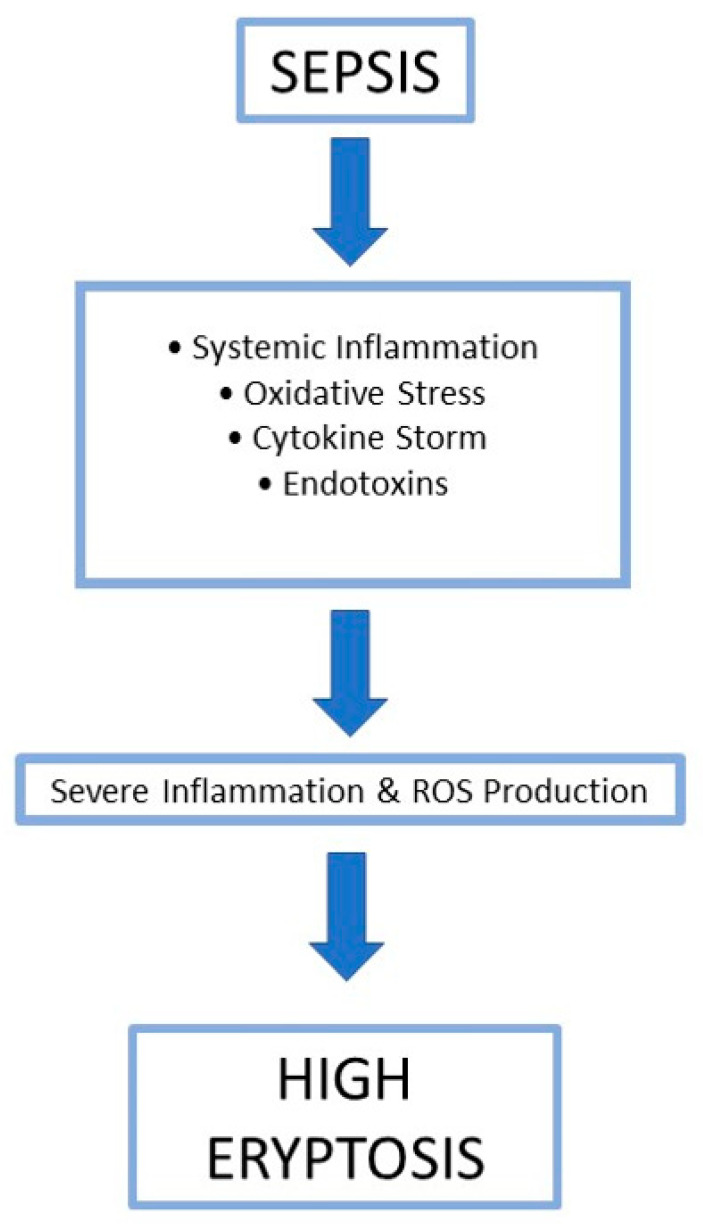
Schematic representation of the mechanisms by which sepsis induces anemia. Sepsis triggers systemic inflammation, oxidative stress, cytokine storm, and endotoxin release, collectively leading to excessive reactive oxygen species (ROS) production and severe inflammation. This environment promotes eryptosis, ultimately resulting in anemia.

Sepsis is characterized by a pronounced, dysregulated, and uncontrolled inflammatory response that significantly contributes to the development of sepsis-induced anemia. During sepsis, excessive immune system activation results in the overproduction and imbalance of pro-inflammatory cytokines and mediators, including tumor necrosis factor-alpha (TNF-α), interleukins 1 and 6 (IL-1, IL-6), and reactive oxygen species (ROS). These factors not only drive systemic inflammation but also impair the bone marrow’s capacity to generate healthy red blood cells, disturb iron metabolism, and reduce the lifespan of circulating erythrocytes. The combined effect of this immune imbalance and oxidative stress triggers premature red blood cell death, known as eryptosis, which further aggravates anemia. As a result, sepsis-induced anemia leads to impaired oxygen delivery to tissues, heightened organ dysfunction, and poorer outcomes in patients with sepsis [11,12,34] (Figure 2).

**Figure 3:**
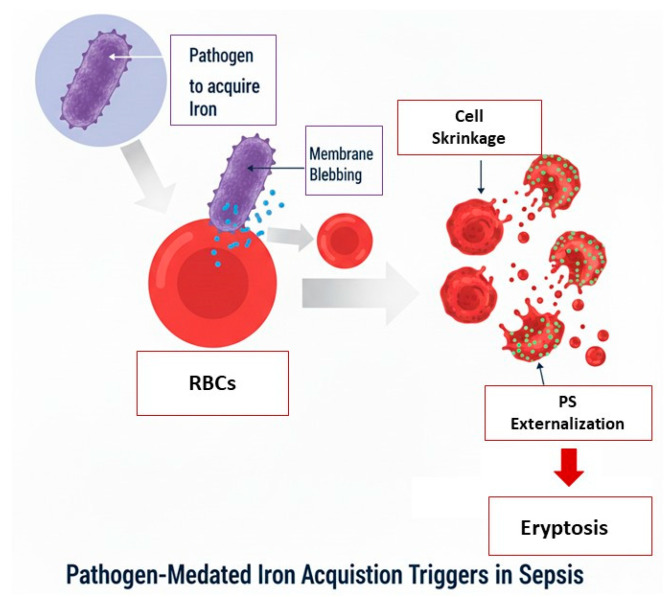
Schematic representation of Pathogen-Mediated Iron Acquisition.

Basically, during the course of the septic infection, pathogens necessitate iron for their survival and proliferation. To acquire it, they may attack erythrocytes triggering modifications to their membrane structure, such as cell shrinkage, membrane blebbing, and the externalization of PS on the cell surface. These alterations finally cause eryptosis (Figure 3).

Consequently, the proportion of damaged erythrocytes increases, leading to a decrease in their overall count and contributing to sepsis-related anemia. The eryptotic red blood cells are subsequently removed from circulation by the reticuloendothelial system, which further worsens the anemic condition in patients with sepsis [11,12,34].

A growing number of in vitro and in vivo studies have recently documented morphological changes in human red blood cells, findings that have been observed both in animal models and in clinical settings. These studies have highlighted features such as reduced cell volume, membrane protrusions, and the externalization of phosphatidylserine: all hallmarks indicative of eryptosis [47,48,49,50,51,52,53] (Table 1). For example, Piagnerelli et al. reported that patients with sepsis showed elevated neuraminidase activity, an enzyme that cleaves sialic acid residues from the erythrocyte surface, thereby altering the membrane’s structural integrity and compromising the rheological properties of RBC [47]. These modifications can reduce the deformability of RBCs and impair their ability to navigate the microcirculation efficiently. Moreover, in a cohort of Brazilian septic patients, significant alterations in red blood cell morphology and shape were observed, primarily as a consequence of oxidative stress. This oxidative damage contributes to membrane instability and further promotes eryptosis, ultimately exacerbating microvascular dysfunction and tissue hypoxia in sepsis [52].

Evidence from studies using animal models has reinforced these observations, demonstrating similar alterations in red blood cell morphology and function under septic conditions. These experimental models have provided valuable insights into the mechanisms underlying erythrocyte damage during sepsis, including increased oxidative stress, enzymatic degradation of membrane components, and accelerated eryptosis. Such findings highlight the translational relevance of animal research in understanding the pathophysiological changes seen in human sepsis. For instance, in a mouse model of sepsis induced by cecal ligation and puncture (CLP), researchers found that extracellular vesicles (EVs) derived from plasma significantly affected RBC deformability, resulting in increased erythrocyte stiffness. The accumulation of EVs in the bloodstream during sepsis may therefore play a pivotal role in modulating RBC function, potentially contributing to systemic complications such as impaired microcirculatory flow and tissue hypoxia [43]. Likewise, in rat models of sepsis induced by CLP, oxidative stress was shown to alter blood rheology by impairing RBC deformability and vascular resistance. These mechanical changes in erythrocytes can compromise microcirculatory flow and reduce the efficiency of oxygen delivery to peripheral tissues, thereby aggravating the systemic impact of sepsis. The reduced flexibility of RBCs caused by oxidative damage plays a key role in the progression of sepsis, affecting both hemodynamic stability and the overall severity of the disease [51].

**Figure 4:**
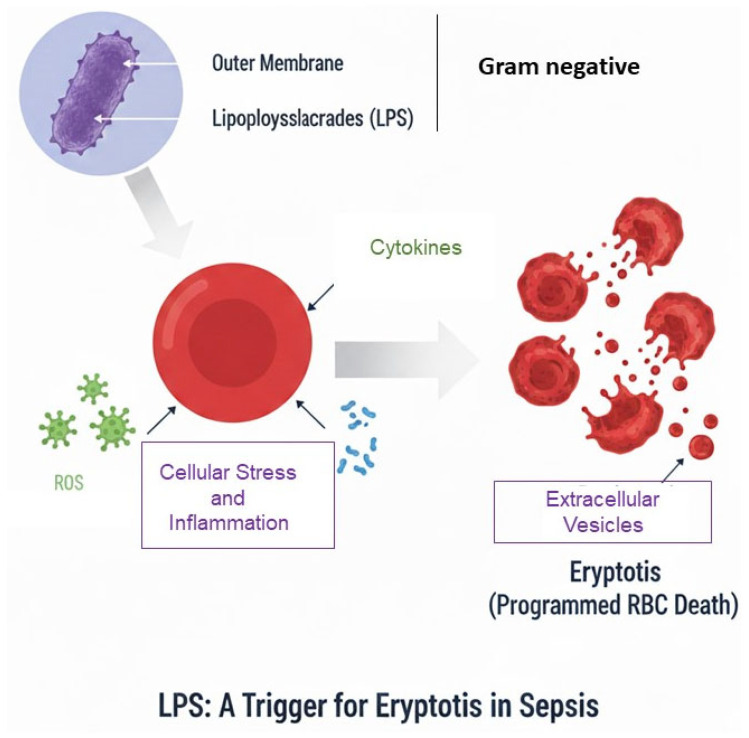
Schematic representation of LPS induction of Eryptosis.

Kempe et al. carried out an experimental study aimed at assessing PS-exposure, RBC volume, intracellular calcium (Ca^2+^) levels, and ceramide generation. These parameters were evaluated using flow cytometry. In their in vitro model, eryptosis was induced in erythrocytes obtained from healthy donors by exposing them to plasma collected from septic patients. The findings demonstrated that sepsis-associated plasma contains factors capable of triggering profound alterations in RBCS, ultimately promoting eryptosis. This underscores the role of circulating mediators in sepsis-related erythrocyte dysfunction [50]. These findings were further validated and expanded upon by Marcello and colleagues, who delved deeper into the molecular mechanisms responsible for eryptosis in the context of sepsis. Their study not only confirmed the pro-eryptotic effect of septic plasma on red blood cells but also identified key signaling pathways and mediators involved in this process. They conducted a combined case–control study incorporating both in vivo and in vitro approaches, with a particular focus on evaluating eryptosis in healthy RBCs exposed to plasma from septic patients over different time intervals. Their research aimed to assess how septic plasma influences the onset and progression of eryptosis and to identify potential temporal patterns in this process. In addition, the study examined the correlation between the degree of eryptosis and several clinically relevant parameters, including Endotoxin Activity Assay (EAA) levels, patient mortality, and a range of biomarkers associated with systemic inflammation and oxidative stress. By integrating these elements, the study provided valuable insights into the prognostic significance of eryptosis in sepsis and highlighted its potential role as a marker of disease severity and outcome [49]. Gram-negative bacterial infections represent a major cause of sepsis and are strongly associated with the development of endotoxemia. The presence of endotoxins, particularly lipopolysaccharides (LPS) found in the outer membrane of Gram-negative bacteria, plays a critical role in triggering profound alterations in the host immune response (Figure 4). These endotoxins activate innate immune pathways, leading to an overwhelming release of pro-inflammatory cytokines, immune cell activation, and ultimately contributing to the dysregulated immune response that characterizes septic states [54].

Recent developments in the field of sepsis diagnostics have made it possible to detect endotoxins directly in whole blood by employing neutrophil-mediated chemiluminescence techniques [55]. In particular, EAA is a chemiluminescent technique used to quickly assess endotoxin levels in patients. High EAA values are considered a significant indicator of severe sepsis and have been strongly associated with greater risk of multi-organ failure and increased mortality rates [56]. This has important diagnostic and therapeutic relevance given the possibility of removing endotoxins from the bloodstream with Extracorporeal Blood Purification Therapy. In the EUPHRATES trial, treatment with a polymyxin-B hemoperfusion cartridge in patients exhibiting EAA levels ranging from 0.6 to 0.89 was associated with a significant reduction in 28-day mortality, after adjusting for APACHE II scores and baseline mean arterial pressure (MAP) [57].

**Figure 5:**
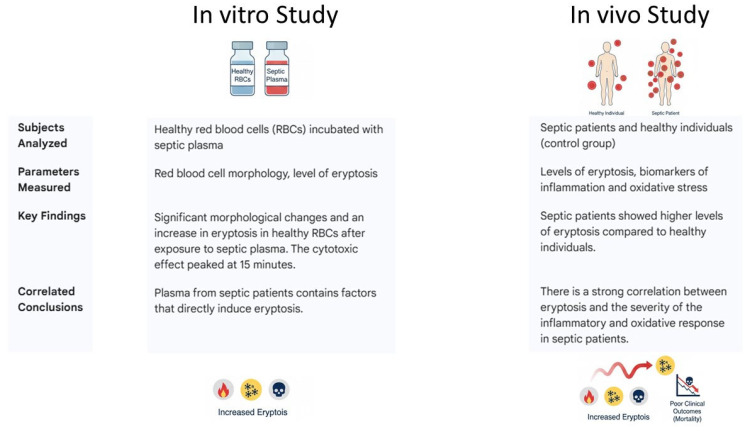
Schematic representation of Marcello and colleagues’ study.

By examining these variables, Marcello and colleagues sought to clarify the connection between septic plasma-induced eryptosis and the extent of inflammatory and oxidative responses, along with its potential impact on clinical outcomes, including mortality. The study revealed significant in vitro findings, showing considerable changes in RBC morphology and an increase in eryptosis in healthy RBCs incubated with septic plasma. Notably, the septic-induced cytotoxic effect peaked at 15 min. In a complementary in vivo study, the same results were observed: septic patients exhibited higher levels of eryptosis compared to healthy individuals. Moreover, the authors found a strong correlation between eryptosis and other biomarkers of inflammation and oxidative stress [49] (Figure 5).

However, no conclusive evidence currently exists to establish a direct link between eryptosis and septic prognosis or disease severity. Additional well-designed prospective studies and mechanistic investigations are needed to clarify these points. Importantly, if the early timing of eryptosis observed in these preliminary studies were to be confirmed by larger and more robust investigations, eryptosis could potentially serve as an early biomarker of sepsis. Its rapid onset compared to other inflammatory and organ-damage markers may also suggest a complementary role, whereby eryptosis measurements could be integrated with established biomarkers to enhance early diagnosis and risk stratification. Several biomarkers and clinical parameters are already employed in the management of sepsis and endotoxemia, including temperature, bacteremia, cytokines, lactate, and indices of organ damage [58,59]. The potential contribution of eryptosis to this existing arsenal lies in its capacity to capture early RBC injury and microcirculatory dysfunction, events that are central to the pathophysiology of sepsis but not fully reflected by conventional biomarkers. By integrating information on oxidative stress, inflammation, and membrane integrity, eryptosis may provide complementary insights into disease progression. Moreover, preliminary evidence suggests that eryptosis may occur at a very early stage in the septic process, raising the possibility of its use as an early biomarker, either alone or in combination with established parameters, to improve diagnostic accuracy, risk stratification, and timely therapeutic decision-making. In this context, the development of a multiparametric diagnostic panel that incorporates eryptosis together with conventional biomarkers could represent an ideal strategy to capture the complex and multifaceted nature of sepsis.

To date, no clinical trials have specifically investigated the role of eryptosis in sepsis, highlighting the need for future studies to explore this potential pathophysiological and therapeutic link.

## 5. Future Directions

**Figure 6:**
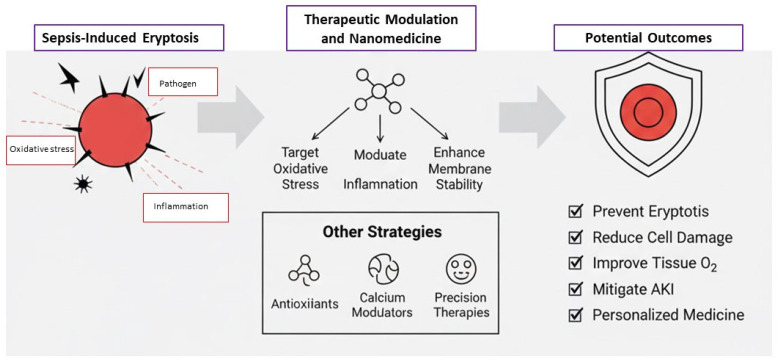
Schematic representation of future directions.

Given the limited, heterogeneous, and largely indirect evidence currently available, future research should prioritize clarifying the mechanistic relevance of eryptosis in both AKI and sepsis. At present, it remains uncertain whether eryptosis constitutes a causal mediator of organ injury or merely reflects the profound systemic inflammation and oxidative stress characteristic of critical illness. High-quality translational studies—integrating cellular models, animal experiments, and well-controlled clinical investigations—are therefore needed to determine whether accelerated erythrocyte death plays an active pathogenic role or represents an epiphenomenon without direct mechanistic impact. In the context of AKI, an important avenue of inquiry concerns the influence of extracorporeal therapies such as CRRT and ECMO. Both modalities expose blood to artificial surfaces, mechanical shear stress, and pro-inflammatory conditions, all of which could theoretically trigger or amplify eryptosis. However, current data remain speculative. Rigorous mechanistic studies are required to assess whether extracorporeal circuits truly promote eryptosis in vivo, whether specific membrane or tubing characteristics exacerbate this process, and whether technical modifications could mitigate erythrocyte injury. Such investigations may also clarify whether eryptosis could serve as an indicator of hemocompatibility in extracorporeal devices—an intriguing but as yet unproven concept. Similarly, in sepsis, key unanswered questions include the temporal pattern of eryptosis activation, the identity of circulating mediators responsible for its induction, and the relationship between endotoxemia, microvascular dysfunction, and red blood cell injury. Integrating eryptosis measurements with markers of inflammation, oxidative stress, and endotoxin activity may help determine whether erythrocyte death possesses prognostic value; however, this hypothesis requires validation through robust clinical studies. The potential role of eryptosis as a biomarker in AKI and sepsis also remains highly speculative. Preliminary observations suggest that eryptosis may appear early in disease, but the paucity of standardized, large-scale evaluations prevents any firm conclusions. Only through systematic, prospective studies will it be possible to determine whether eryptosis meaningfully contributes to early diagnosis, risk stratification, or monitoring of clinical trajectories in critically ill patients. Emerging therapeutic strategies—such as nanoparticle-based agents, antioxidants, calcium-channel modulators, or erythrocyte membrane–stabilizing compounds—may offer future opportunities to modulate eryptosis. However, given the absence of compelling evidence supporting a causal role of eryptosis in organ dysfunction, such interventions should currently be viewed as experimental and justified only within hypothesis-driven frameworks. Likewise, modifying extracorporeal circuits to reduce hemolytic stress represents an attractive concept, but its clinical impact remains to be demonstrated. Overall, significant knowledge gaps continue to limit the interpretation and translational applicability of existing studies. Whether eryptosis is a pathogenic driver, a bystander, or a compensatory response in AKI and sepsis remains unresolved. Addressing these uncertainties will require coordinated efforts employing in vitro models, animal experiments, and well-designed clinical studies aimed at testing explicit mechanistic hypotheses. Only through such rigorous investigation can the true clinical relevance of eryptosis—and its potential role as a biomarker or therapeutic target—be established. Furthermore, understanding the molecular triggers of eryptosis in the septic milieu may allow the development of precision therapies that selectively target these pathways, ultimately contributing to personalized treatment approaches aimed at reducing morbidity and mortality in critically ill patients [38] (Figure 6).

We expect that this field will be increasingly developed in future research through a combination of animal studies, in vivo investigations, and in vitro experimental models designed to clarify the mechanistic pathways and identify effective therapeutic interventions, and evaluate the utility of eryptosis as a biomarker for early diagnosis and multiparametric patient assessment.

The current understanding of eryptosis is hampered by several significant limitations, as highlighted in the text. The evidence currently available is limited and largely indirect, making it difficult to definitively establish the causal role of eryptosis in the pathogenesis of AKI and sepsis. In the absence of robust translational and clinical studies, it remains unclear whether eryptosis represents a true cause or merely an epiphenomenon of systemic inflammation and oxidative stress. These knowledge gaps currently limit the immediate use of eryptosis as a routine biomarker or a validated therapeutic target.

## 6. Conclusions

In conclusion, the available evidence—although limited, fragmented, and largely preclinical—suggests that eryptosis may play a contributory role in the development and worsening of acute kidney injury (AKI), particularly in the context of sepsis. Rather than presenting established mechanisms, we propose a working hypothesis: that the accelerated red blood cell (RBC) death observed during severe systemic inflammation and oxidative stress could foster a pathological cascade marked by anemia, impaired tissue oxygenation, and microcirculatory dysfunction, ultimately aggravating renal damage in critically ill patients.

However, this hypothesis remains constrained by substantial gaps in experimental and clinical evidence. The precise pathways linking eryptosis to AKI progression are still incompletely understood, causality has not been demonstrated, and most supporting data derive from heterogeneous models with limited translational applicability. These weaknesses must be acknowledged as major limitations and underscore the speculative nature of the proposed framework.

Despite these uncertainties, exploring eryptosis as a potential pathogenic factor—and possibly a biomarker—could open new avenues for research in sepsis-associated AKI (S-AKI). Future studies are urgently needed to validate whether eryptosis contributes meaningfully to renal injury, to clarify its mechanistic relevance, and to determine whether its modulation could represent a viable therapeutic or diagnostic strategy. A rigorous, hypothesis-driven research agenda may ultimately help assess whether targeting eryptosis can improve risk stratification, personalize supportive therapies, and potentially influence outcomes in critically ill patients.

## Figures and Tables

**Table 1:** Key studies and their findings regarding the impact of sepsis on RBCs.

Study	Year	Organism	Model/System	Key Findings	Impact on RBCs
Piagnerelli et al.	2009	Human	Septic patients	Enhanced neuraminidase activity observed in sepsis patients alters red blood cell rheological properties.	Modified RBC rheological properties
Oliveira et al.	2017	Human	Septic patients	Oxidative stress induces marked changes in red blood cell morphology and structural characteristics.	Changes in RBC morphology and structural shape
Subramani et al.	2018	Mouse	CLP-induced sepsis	The presence of plasma-derived EVs adversely affects RBC deformability, resulting in enhanced rigidity.	Elevated red blood cell stiffness accompanied by decreased deformability.
Bostanci et al.	2018	Rat	CLP-induced sepsis	RBC deformability decreases and resistance increases under oxidative stress.	Reduced red blood cell deformability causes microcirculatory dysfunction and impaired oxygen transport.
Kempe et al.	2007	Human	In vitro model: Healthy RBCs exposure to septic plasma	Septic patient plasma triggers eryptosis through increased PS exposure, RBC swelling, cytosolic calcium, and ceramide accumulation	Triggered eryptosis in RBCs
Marcello et al.	2023	Human	In vivo and in vitro models: septic patients and healthy RBCs exposure to septic plasma	Investigation of eryptosis induction alongside measurement of EAA levels, mortality rates, and markers of inflammation and oxidative stress.	Induction of eryptosis, notable changes in red blood cells, and correlations with EAA concentrations and relevant biomarkers.

## Data Availability

No new data were created or analyzed in this study. Data sharing is not applicable to this article.
